# Composition-Dependent Interlayer Stability and Oxygen-Vacancy Formation in Ni–Co–Mn Layered Oxides: Implications for Lithium-Ion Battery Cathode Recycling

**DOI:** 10.3390/ma19214494

**Published:** 2026-10-26

**Authors:** Bekzat Zhumabay, Danil W. Boukhvalov, Yerlan O. Koptleuov, Nikolay A. Chuchvaga

**Affiliations:** 1 Institute of Physics and Technology, Satbayev University, Almaty 050032, Kazakhstan; b.zhumabay@satbayev.university; 2 Institute of Materials Physics and Chemistry, College of Science, Nanjing Forestry University, Nanjing 210037, China; 3 Institute of Energy and Mechanical Engineering, Satbayev University, Almaty 050043, Kazakhstan; kerlan207@gmail.com

**Keywords:** lithium-ion batteries, cathode recycling, layered oxides, Ni–Co–Mn oxides, density functional theory, oxygen vacancies, delamination, residual lithium

## Abstract

Layered transition-metal oxides are key cathode materials for lithium-ion batteries and promising feedstocks for direct recycling. Their recycling behavior is strongly governed by oxide-framework stability, interlayer interactions, defect formation, and residual lithium content. In this work, spin-polarized density functional theory calculations were employed to investigate the composition-dependent structural stability of Mn- and Co-doped NiO_2_-based systems. The Co and Mn contents were systematically varied to evaluate interlayer distances, interlayer binding energies, oxygen-vacancy formation energies, and the influence of residual lithium. The results indicate that transition-metal composition strongly affects both the interlayer coupling strength and the energetic preference for oxygen-vacancy formation. In particular, compositional changes modify the balance between structural integrity and layer separability, while residual lithium further alters the stability of the layered framework. These findings clarify the roles of cation chemistry and defect formation in governing the propensity for delamination in spent layered cathodes. The results provide atomistic design principles for selecting recycling routes, direct reduction, or liquid-phase delamination for the studied cathode materials and may support the development of more efficient, composition-specific recycling strategies.

## 1. Introduction

Recycling of Li-ion batteries (LIBs) is an emerging topic in modern applied sciences [1]. Experimental work has unambiguously demonstrated that LIB cathodes consist of MO_2_ (where M = Ni, Co, Fe, and Mn) layers separated by ordered lithium layers [2,3]. After lithium extraction, two scenarios are possible: the first is the collapse of a layered system into a 3D crystal, and the second is the survival of layered structures [4,5]. The latter scenario is very promising for recycling because preliminary delamination can facilitate reduction. Note that the reduction of layered cathode materials can be achieved not only in metals but also in layered double hydroxides, which are emerging materials with multiple prospective applications [6,7]. Thus, knowledge of interlayer distances and chemical bonds is essential for improving the efficiency of LIB recycling. Beyond practical importance, several fundamental questions are raised. One of these questions is the nature of the interlayer bonds in layered oxides. Another fundamental question is the effect of “typical” and “atypical” oxidation states on the stability of layered oxides and the energetics of oxygen-vacancy formation.

Density functional theory (DFT)-based methods demonstrate significant potential for exploring layered structures and revealing different types of interlayer non-covalent bonds [8]. Recent studies have mainly considered the effect of the chemical composition of MO_2_ layered structures on lithium or sodium capacity and voltage (see Z. Wand et al. [9] for a review). The effect of chemical composition on lithium migration has been considered in many studies [10,11,12]. Another set of studies examined the effects of defects and surface states on the migration of different ions [9,13].

Recent simulations of layered oxide cathodes have therefore evolved from equilibrium crystal-structure calculations toward multiscale descriptions of degradation and regeneration. At the atomistic scale, density functional theory (DFT)—especially DFT+*U*—meta-GGA approaches such as SCAN, and hybrid-functional benchmarks are used to examine cation redox, Li-vacancy ordering, oxygen stability, migration barriers, phase competition, and surface reactivity. The correct treatment of transition-metal 3*d* states is particularly important because the predicted oxidation states, band structure, Li-extraction energetics, and propensity for cation disorder are highly sensitive to the electronic-structure approximation. Recent methodological work has shown that more physically realistic correlated-orbital projectors can improve DFT+*U* predictions of lattice parameters, electronic configuration, and oxidation-state assignment in LiCoO_2_, LiNiO_2_, and Ni–Co–Mn layered oxides [14].

Thus, previous DFT-based studies of layered oxides have focused on identifying a chemical composition that maximizes LIB performance. The effects of chemical composition and residual lithium on recycling efficiency have not been studied. In this work, we report a systematic study of how gradual changes in the chemical composition of NiCoMnO_2_-based systems affect vacancy formation energies, interlayer distance, and binding energies. The effects of oxygen vacancies and residual lithium on the stability of NiCoMnO_2_-based systems are also presented. We discuss the potential for recycling via direct reduction or liquid-phase delamination in MO_2_-based materials with different chemical compositions.

## 2. Computational Method

We performed theoretical modeling using the QUANTUM-ESPRESSO pseudopotential code [15], employing the generalized gradient approximation [16] for the exchange-correlation potential in spin-polarized mode. A full optimization of atomic positions and lattice parameters was carried out. During optimization, the electronic ground state was consistently found using ultrasoft pseudopotentials [17]. The contributions from van der Waals forces were taken into account [18]. The cut-off energy was set to 35 Ry for all calculations. A *k*-point mesh of 6 × 6 × 3 for bulk and 6 × 6 × 1 for monolayers was used in the Monkhorst–Pack scheme [19]. To plot the final densities of states (DOSs), the *k*-point mesh was increased to 8 × 8 × 4. For all calculations, one-site Coulomb repulsion was applied to all 3*d* orbitals [20]. The values of U and J are 4 eV and 0.9 eV, respectively, according to the work by Y.-Y. Ting et al. [14]. The forces and total energies were optimized with accuracies of 0.04 eV Å^−1^ and 1.0 meV/supercell, respectively.

## 3. Results and Discussion

### 3.1. The Nature of Interlayer Bonds in Transition-Metal Dioxides

**Figure 1:**
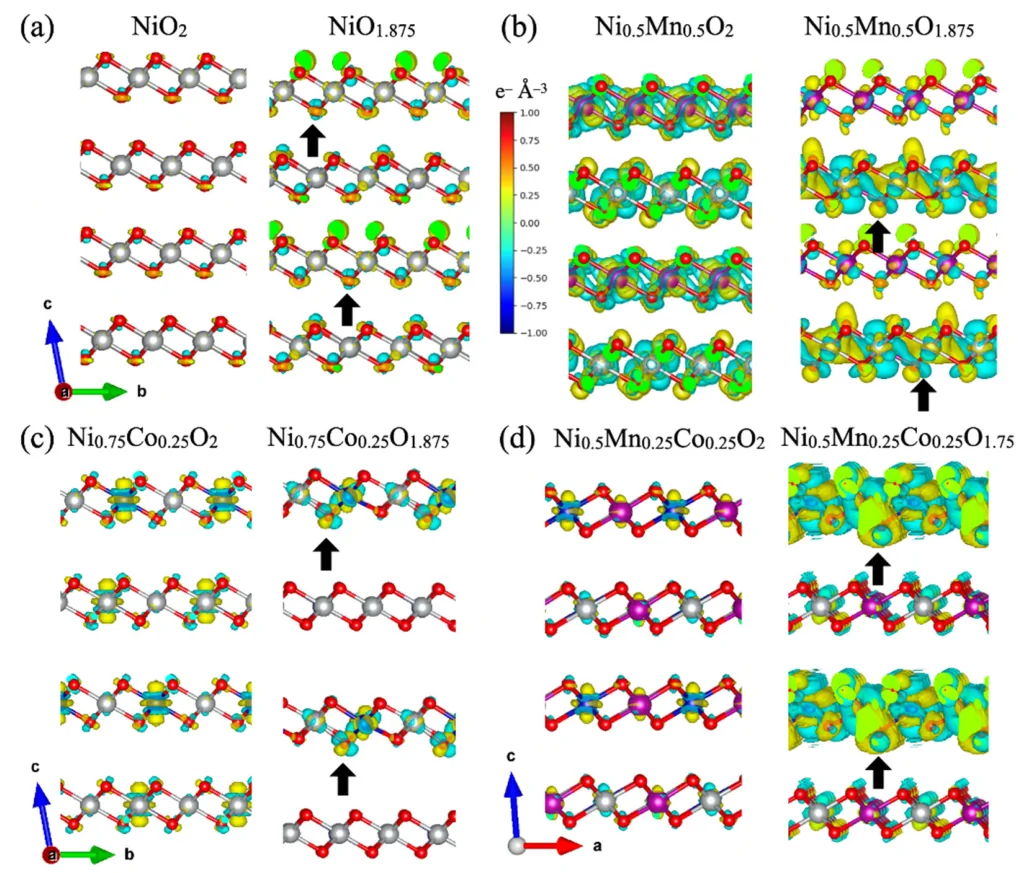
Charge-density redistributions for the formation of bulk NiO_x_ (**a**), Ni_0.5_Mn_0.5_O_x_ (**b**), Ni_0.75_Co_0.25_O_x_ (**c**), and Ni_0.5_Mn_0.25_Co_0.25_O_x_ (**d**) from monolayers. The values of isosurface layers are the same for all panels (0.005 e^−^ Å^−3^). Nickel atoms are shown in red, manganese in violet, cobalt in dark blue, and oxygen in red. Yellow “clouds” correspond to an increase in charge density and cyan clouds correspond to a decrease (see color scale). The locations of the oxygen vacancies are marked by thick arrows.

**Figure 2:**
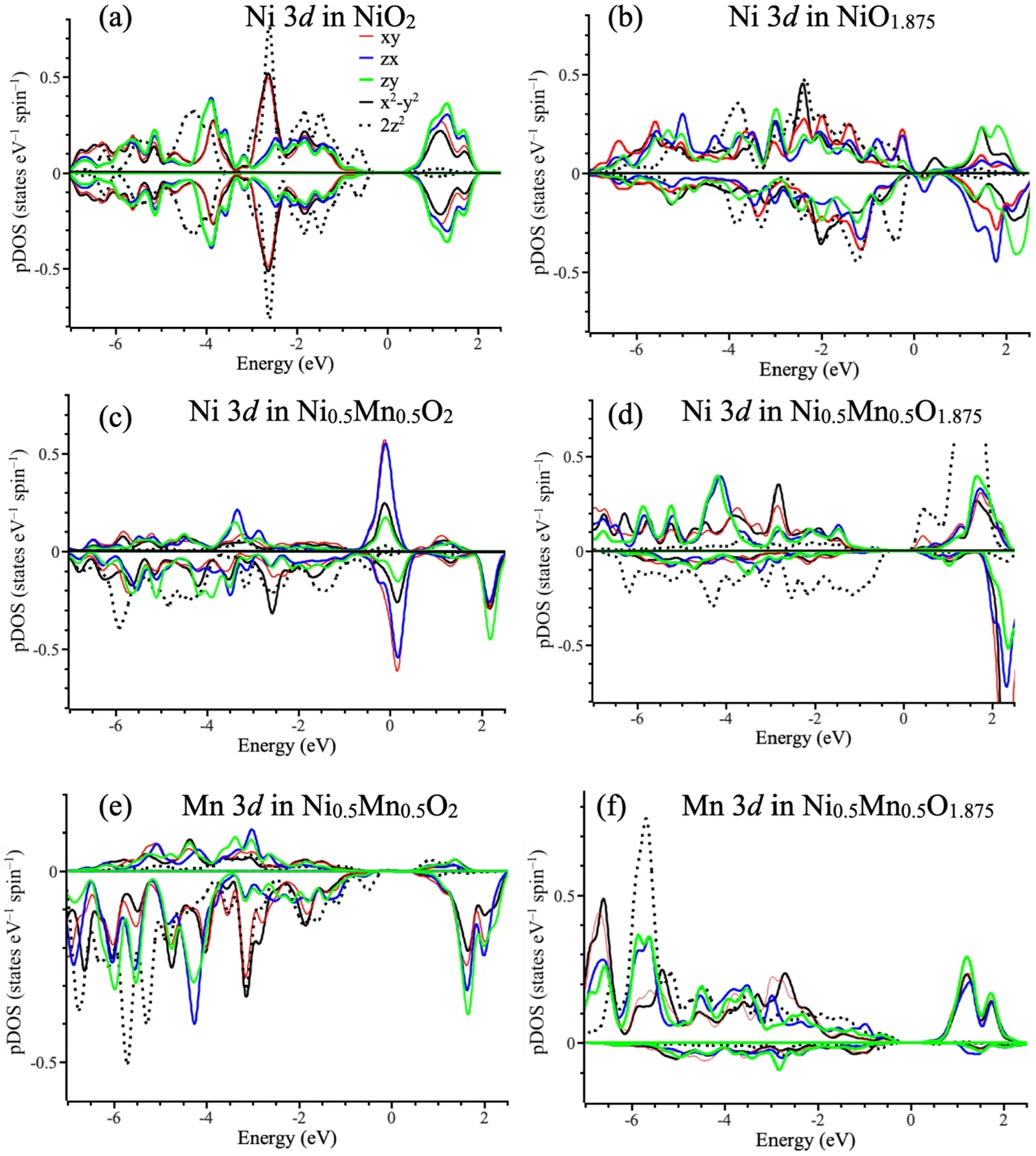
Orbital-resolved spin-polarized densities of states for 3*d* orbitals of nickel in NiO_x_-based systems (**a**,**b**) and Ni_0.5_Mn_0.5_O_x_-based (**c**,**d**) systems, and 3*d* orbitals of Mn in Ni_0.5_Mn_0.5_O_x_-based (**e**,**f**) systems. For reduced systems, the electronic structure is shown for the metal centers near vacancies.

Based on the literature, NiO_2_ layered structures (Figure 1a) have been chosen as the starting model [3,21]. Each unit cell contains two layers in a *1T* configuration of the AB structure [6,7,14]. For our modeling, we used a 2 × 2 × 1 supercell within periodic boundary conditions. This supercell contains eight metal centers (four in each layer); thus, each step in the chemical composition corresponds to 12.5 at.%. This supercell size balances the computational cost of simulating multiple atomic structures with a minimal step size for varying chemical composition. Our previous work demonstrates that the analysis of differential charge density is a powerful tool for describing interlayer bonds in layered structures [8]. In defect-free NiO_2_, the transition from monolayers to layered bulk structures corresponds to insignificant changes in the charge density (Figure 1a). This pattern and magnitude of charge redistribution are similar to those calculated by a similar method for graphene and hBN (0.006 e^−^ Å^−3^ vs. 0.003 e^−^ Å^−3^) and are much smaller than those reported for the systems with dipole–dipole interaction-based interlayer bonds [8]. Thus, we conclude that interlayer bonding in these systems is attributable solely to London dispersion forces [22]. The absence of a dipole moment in the NiO_2_ layer can be attributed to the low-spin configuration of six-coordinated Ni^4+^ (zero magnetic moment). Because layered systems are anisotropic, electric polarization of the layers is associated with in-plane (xy and x^2^–y^2^) and out-of-plane (xz, yz, and especially 2z^2^) orbital occupancies in the 3d shell of metal ions. Partial density-of-states plots show that this configuration corresponds to an almost uniform distribution of electrons in space (Figure 2a), consistent with the absence of permanent polarization.

Forming an oxygen vacancy in one layer of the supercell leads to more significant charge redistribution upon the monolayer-bulk transition (Figure 1a). Note that this charge redistribution is more pronounced in the layer with an oxygen vacancy. In other words, the oxygen vacancy polarizes the layer, and the layer’s electric field polarizes the other layer. The cause of this charge redistribution is the reduction of two nickel atoms near the vacancy. This reduction corresponds to a turn of the magnetic moment from zero to 0.41 μ_B_ and significant changes in the electronic structure of these atoms (Figure 2b). The most significant changes in the area near the Fermi level were observed for out-of-plane xz and 2*z^2^* orbitals (represented by solid blue and dashed black lines in Figure 2b). These local changes in electronic structure are the cause of the layer’s polarization. Thus, interlayer interactions in NiO_1.875_ can be described as dipole-induced dipole interactions [22].

Substitution of half of the nickel atoms by manganese leads to a significant charge redistribution upon monolayer-bulk transition (Figure 1b). Reduction of one of the layers decreases the magnitude of charge redistribution in the reduced layer; however, the charge redistribution is more significant than in NiO_1.875_ (Figure 1b vs. Figure 1a). Thus, we can propose that Mn-doping induces dipole–dipole interactions between NiOx-based layers. Note that, in contrast to Ni^4+^-based compounds, natural Mn^4+^-based compounds are numerous. Manganese centers in these compounds always have non-zero magnetic moments due to an odd number (three) of electrons in the 3*d* shell. In Ni_0.5_Mn_0.5_O_2_, the magnetic moment on manganese centers is 1.44 μ_B_, whereas it is zero on nickel centers. The non-uniform distribution of electrons between in-plane and out-of-plane shells corresponds to electric polarization in the layer (Figure 2c). On the contrary, the formation of oxygen vacancies turns Mn^4+^ to Mn^3+^ with an even number of electrons in the 3*d* shell, which corresponds to changes in electronic structure and a decrease in magnetic moments (from 1.44 μ_B_ to 0.79 μ_B_) and in the polarity of the reduced layer (Figure 1b and Figure 2d).

Substituting a quarter of the nickel atoms with cobalt forms a weak dipole–dipole interaction between the layers. Oxygen vacancies induce the transition of Co^4+^ to Co^3+^ and reduce the magnetic moment at cobalt centers from 0.9 μ_B_ to 0.57 μ_B_. These changes in the electronic structure of cobalt make the reduced layer non-polar. The absence of electron-charge-density redistribution in vacancy-free layers, combined with only local charge-density redistribution near the oxygen vacancy in the reduced layer (see right subpanel in Figure 1c), means the dipole moment on the unreduced layer is insufficient to induce polarization in the non-polar layer. The combination of Co and Mn impurities in NiO_2_ corresponds to dipole–dipole bonds between the layers. The magnitude of charge-density redistribution upon monolayer-to-bulk redistribution is smaller than that in Mn-doped NiO_2_ but of the same order of magnitude as that in Co-doped NiO_2_ (Figure 1c,d). Reducing one layer increases the magnitude of charge-density redistribution upon the monolayer-to-bulk transition, thus enhancing dipole–dipole interactions. Since reducing the layer switches the spin on the manganese center near the vacancy without qualitative changes in electronic structure (Figure 2e,f), these changes in charge-density redistribution can be attributed solely to changes in reduced nickel centers.

Based on these results, we conclude that the dominant interlayer bonding in MO_2_-based systems is electrostatic bonding. The combination of chemical composition and oxygen vacancies can influence the strength of these interactions by altering dipole–dipole interactions or even shifting interlayer interactions toward dipole-induced dipole and London dispersion-based interactions. The type and strength of interlayer bonds are defined by redistribution of electron density among different orbitals in 3*d* shells of metal ions. Observed changes in charge density and interlayer distance are aligned with experimentally observed effects of transition-metal doping on Ni-rich cathodes [23]. The local combination of metal anions and oxygen vacancies determines interlayer-bond strength and, therefore, the possibility of layer exfoliation. The effect of chemical composition on interlayer distances and bond strength is considered in Section 3.2.

### 3.2. Effect of Chemical Composition on Lattice Stability

Based on visible changes in differential charge densities, we systematically screened interlayer distances, binding energies, and oxygen-vacancy formation energies for MO_2_-based layered structures with different chemical compositions. Based on results reported in the previous section, Mn and Co dopants in NiO_2_ layers were considered representative for a comprehensive description of the effect of impurities. The effect of oxygen vacancies in one of the layers was also considered. Because our model unit cell includes two layers, oxygen vacancies and defects are distributed between layers in a non-equivalent manner; we define the interlayer distance as the size of the two-layer supercell along the vertical axis. Binding energy is defined as the energy required to delaminate the bulk.

(1) E_bind_ = E(layer1) + E(layer2) − E(bulk)

In this definition, large positive values correspond to very stable structures, and negative binding energies correspond to spontaneous delamination. For all bulk and monolayers, a full optimization of atomic positions and lattice parameters was performed. This mode of calculation permits taking into account possible expansion or shrinking of monolayers upon exfoliation.

**Figure 3:**
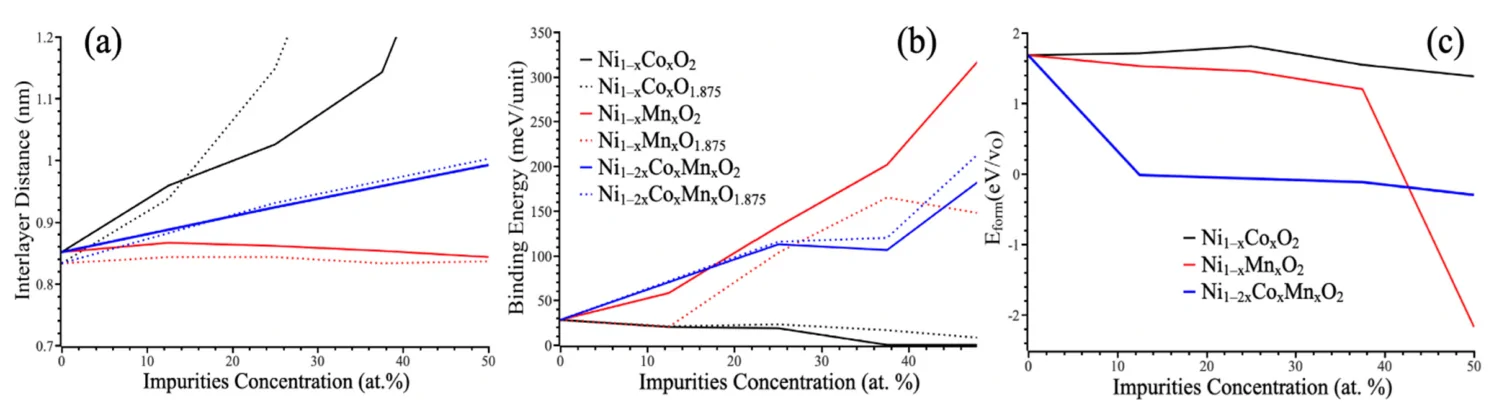
Interlayer distance (**a**), binding energy (**b**), and oxygen-vacancy formation energy (**c**) as a function of Co and Mn impurity concentration in NiO_2_-based layered structures. Note: Since an odd number of defects (dopants and oxygen vacancies) in a supercell is unevenly distributed between the two layers, we considered the distance between two equivalent layers under periodic boundary conditions as the interlayer distance.

First, we examined the effects of Mn and Co doping on interlayer distances and binding energies by varying the concentrations of cobalt and manganese impurities in NiO_2_ layers. The binding energy in pristine NiO_2_ is 28 meV/unit, close to values calculated by similar methods for graphene and hBN (17 and 16 meV/unit, respectively) [8], supporting the description of interlayer bonds as arising from London dispersion forces. For all studied materials, insignificant decreases of 0.5~2% in the in-plane lattice parameters were observed (in bulk, in-plane lattice parameters are equal to 0.288~0.296 nm; in the monolayer, they shrink to 0.280~0.291 nm). Note that these changes are much smaller than the changes in interlayer distances upon doping and the introduction of oxygen vacancies (Figure 3a). In the case of cobalt doping, interlayer distances increased even at the lowest concentrations; this occurred simultaneously with a fast decrease in binding energies (see black solid lines in Figure 3a,b). A visible increase in interlayer distance in Ni_x_Mn_1−x_O_2,_ even after a significant increase in binding energy, results from steric repulsion between layers that inhibits further approach. In contrast, the decrease in binding energy with increasing cobalt content is not limited by steric repulsion and, therefore, corresponds to spontaneous delamination when cobalt concentration exceeds 25%. The described changes in binding energies align with the changes in charge densities discussed in the previous section.

Co-doping of NiO_2_ with equal amounts of cobalt and manganese presents a less straightforward picture: a simultaneous increase in binding energy and interlayer distance (represented by the blue lines in Figure 3a,b). The increase in binding energy can be attributed to a larger change in binding energy caused by manganese doping than by cobalt doping (see red vs. black lines in Figure 3b). On the contrary, for the interlayer distance effect, cobalt is stronger than manganese (see red vs. black lines in Figure 3c). Thus, changes in interlayer distance and binding energies can be described as a superposition of dopant contributions. Because the increase in Co content leads to weakening of interlayer bonds and an increase in interlayer distances in oxygen-rich and oxygen-poor NiO_2_-based systems (Figure 3a,b), we propose that liquid-phase delamination is more favorable for Co-rich cathode materials. Note that reported values provide only a qualitative picture since exact reduction energies depend on surface morphology and the solvent used. However, recent experimental studies demonstrate a similar effect of cobalt on the exfoliation process in similar systems [24,25].

In the previous paragraphs, we approached recycling from an exfoliation perspective. Another approach to recycling cathode materials is direct reduction by oxygen extraction [1]. The exact energy cost of the reduction depends on the chemical composition and morphology of the reducing agent. The relative energy cost is sufficient to describe the effect of chemical composition on MO_2_-based layer reduction. The formation energies for oxygen vacancies have been calculated by the standard formula:

(2) E_form_(v_O_) = E(MO_1.875_) + 1/2E(O_2_) − E(MO_2_),

where E(O_2_) is the energy of an oxygen molecule in the gaseous phase. Negative formation energies indicate spontaneous reduction. Since, in the real world, oxygen migrates from MO_2_ layers to the substrate during oxidation, reported values of E_form_(v_O_) provide only a relative indication of the tendency of MO_2_ layers to be reduced.

Cobalt doping does not lead to significant changes in the oxygen-vacancy formation energies across all studied impurity concentrations (see the black lines in Figure 3c). On the contrary, manganese doping destabilizes the Ni_1−x_Mn_x_O_2_ lattice even at the lowest manganese content (12.5 at%; see the blue line in Figure 3c). Co-doping of Ni_1−x_Mn_x_O_2_ with cobalt stabilizes the lattice: the decrease in oxygen-vacancy formation energy occurs at 37.5 at% of impurities (see the red line in Figure 3c). The formation of the oxygen vacancy turns local M^4+^O_6_ to M^3+^O_5_ (where M is a metal ion). The latter structure is more favorable for manganese than for nickel, and especially cobalt [26]; therefore, manganese reduces the oxygen-vacancy formation energy. The stabilizing effect of cobalt, associated with an increased energy cost for oxygen-vacancy formation, can be attributed to the lower stability of the five-coordinated Co^3+^. Thus, Mn-rich cathodes can be utilized by direct reduction. Note that reported values provide only a qualitative picture, since exact reduction energies depend on surface morphology, reaction kinetics, and the reducing agent.

### 3.3. Effect of Remaining Lithium on Lattice Stability

**Figure 4:**
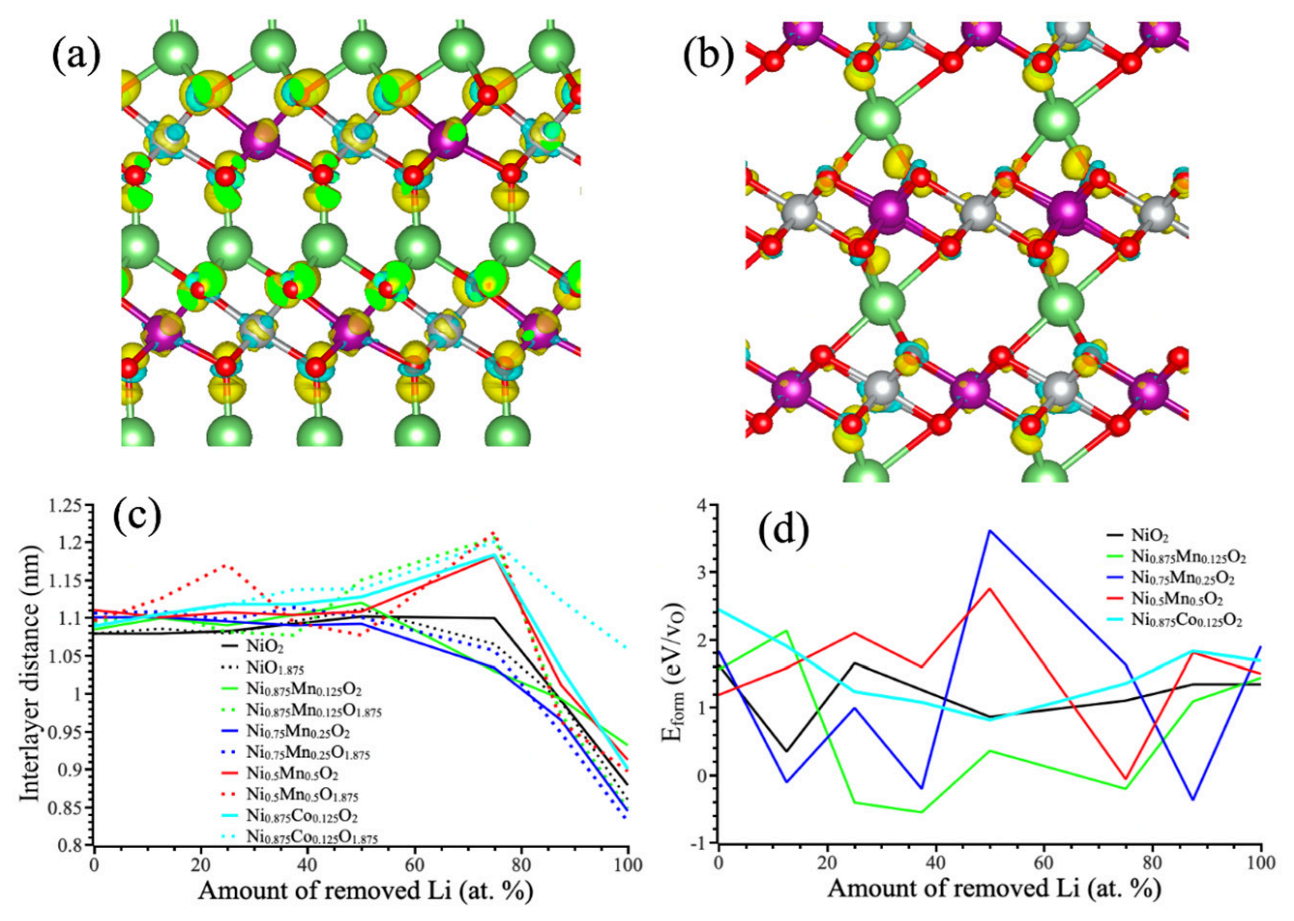
Optimized atomic structure and charge-density redistribution of Ni_0.5_Mn_0.5_O_2_ with maximal load of lithium (**a**), and after removal of 75% of lithium (**b**). Lithium atoms are shown in green, manganese in violet, cobalt in gray, and oxygen in red.The values of isosurface layers are the same for all panels (0.01 e^−^ Å^−3^). The charge-density color scale is the same as in Figure 1. Interlayer distance (**c**) and oxygen-vacancy (v_O_) formation energies (**d**) are displayed as a function of lithium content. Note: Since an odd number of defects (dopants and oxygen vacancies) in a supercell is unevenly distributed among the two layers, we considered the distance between two equivalent layers in periodic boundary conditions as the interlayer distance.

Experimental work demonstrates the presence of residual lithium in recycled cathode materials (see J. Castro et al. and the references therein [27]). Therefore, the final step of our work is to assess the effect of interlayer lithium on interlayer distances and the energy cost of oxygen-vacancy formation. For this purpose, we simulated the maximum lithium load in several representative NiO_2_-based systems and then gradually reduced the lithium content (see Figure S1 and Figure 4a,b). For all systems, the simulation of oxygen vacancies in one layer was performed. In the previous section, we showed that manganese reduces interlayer distances and, conversely, destabilizes the crystal structure of NiO_2_-based systems due to oxygen loss. Therefore, in this section, we focus on Mn-doped NiO_2_ and use Ni_0.875_Co_0.125_O_2_ as a reference.

First, we calculated the effect of the gradual extraction of lithium from selected hosts. Calculations show that for almost all studied systems, extracting less than 75% of lithium does not produce a visible increase in interlayer distance (Figure 4c). The next step of extraction leads to a substantial (about 20%) increase in interlayer distance in Ni_0.5_Mn_0.5_O_2_- and Ni_0.875_Co_0.125_O_2_-based systems, as well as in Ni_0.875_Mn_0.125_O_1.875_. This increase in distance results from the transformation from the structure shown in Figure 4a to the structure shown in Figure 4b. Extraction of all remaining lithium caused the lattice to return to its initial structure, as shown in Figure 1. Interlayer distances for all systems are the same as reported in Figure 3a. This result aligns with the experimentally observed stability of NiO_2_-based systems over many charging cycles [5,23]. Based on these simulations, we propose that a certain amount of residual lithium benefits the intercalation and exfoliation of Mn-rich NiO_2_-based cathode materials.

The final step of our work is a study of the effect of remaining lithium on oxygen-vacancy (v_O_) formation energy. The calculated numbers are shown in Figure 4b. For all studied systems, the line bends correspond to alternating even–odd numbers of lithium atoms in the supercell. For pure and cobalt-doped NiO_2_, the energy cost of oxygen extraction is close to that calculated for Li-free systems (see Figure 3c). For Mn-doping below 25%, the formation energy decreases to values close to zero, even at a high lithium content. At higher manganese content, reduction is favorable only at a low lithium content (25%). This can be attributed to a lower amount of MO_2_-layer doping from lithium (see changes in yellow “clouds” on metal centers in Figure 4a,b). Since in many industrial NiO_2_-based cathodes the manganese content is 10~20% [23,27], a certain amount of residual lithium is beneficial for the practical reduction of batteries. Recent experimental studies also demonstrate a positive effect of residual lithium on recycling Ni-rich cathode materials [27,28,29].

## 4. Conclusions

This work presents a systematic first-principles investigation of layered Ni–Co–Mn oxide systems relevant to the recycling of lithium-ion battery cathodes. Using DFT calculations, we evaluated how gradual changes in transition-metal composition affect the interlayer geometry, interlayer binding, oxygen-vacancy formation, and the stability of lithium-containing and lithium-depleted structures.

The calculations show that the chemical identity and concentration of Ni, Co, and Mn are key factors governing cohesion between adjacent oxide layers. Changes in cation composition modify interlayer distance and binding energy, influencing whether a layered oxide is more likely to retain its structural integrity or separate during recycling. Cobalt most strongly reduces binding energy and increases interlayer distance. Thus, delamination is a preferable route for recycling Co-rich cathode materials.

The composition dependence of oxygen-vacancy formation energies further indicates that defect generation is not a universal property of NiCoMnO_2_-type cathodes; instead, it must be considered alongside the local transition-metal environment. Usually, Mn doping decreases oxygen-vacancy formation energy; cobalt impurities do not affect the reduction of NiO_2_-based systems. Thus, reduction of the cathode materials is preferable in Mn-rich systems.

Residual lithium also plays an important role in stabilizing the layered framework and in changing the energetic conditions for structural transformation. Consequently, the delithiation state of spent cathode material should be considered when selecting a recycling route. Materials with comparatively weak interlayer coupling (especially cobalt-rich NiO_2_) may be particularly suitable for liquid-phase delamination followed by chemical processing, whereas compositions with more strongly bound layers may favor direct reduction-based routes.

Overall, the results establish a direct link between transition-metal composition, defect chemistry, and the recycling-relevant stability of layered MO_2_ cathodes. These findings provide an atomistic basis for composition-tailored recycling strategies and motivate future studies that include explicit solvent effects, reconstructed surfaces, larger defect concentrations, cation disorder, and experimentally representative partially delithiated states.

## Data Availability

The raw data supporting the conclusions of this article will be made available by the authors on request.

## Supplementary Materials

The following supporting information can be downloaded at: https://www.mdpi.com/article/10.3390/ma19214494/s1, Figure S1. Optimized atomic structure of the NiO_2_ supercell at different Li loadings, and data sets for Figure 3; Table S1: Dataset for Figure 3a; Table S2: Dataset for Figure 3b; Table S3: Dataset for Figure 3c. Table S4: Dataset for Figure 4c; Table S5. Dataset for Figure 4d.

## Abbreviations

The following abbreviations are used in this manuscript:

LIB	Lithium-ion battery
DFT	Density functional theory
MO	Metal oxide
