# Supplementary material for: Composition-Dependent Interlayer Stability and Oxygen-Vacancy Formation in Ni–Co–Mn Layered Oxides: Implications for Lithium-Ion Battery Cathode Recycling
Source: Materials (Basel). 2026 Oct 26;19(21):4494. doi: 10.3390/ma19214494 (PMC13764502; doi:10.3390/ma19214494)
Supplement: Supplementary file 1 [file materials-19-04494-s001.zip › materials-4544177-supplementary.pdf]

Supplementary information for the paper

Composition-Dependent Interlayer Stability and Oxygen-Vacancy Formation in Ni–Co–Mn Layered Oxides: Implications for Lithium-Ion Battery Cathode Recycling

Bekzat Zhumabay [1], Danil W. Boukhvalov [1,2,*], Yerlan O. Koptleuov [3] and Nikolay A. Chuchvaga [2]

[1] Institute of Physics and Technology, Satbayev University, Almaty 050032, Kazakhstan;

[2] College of Science, Institute of Materials Physics and Chemistry, Nanjing Forestry University, Nanjing 210037, P. R. China;

[3] Institute of Energy and Mechanical Engineering, Satbayev University, 22 Satbaev St., Almaty, 050043, Kazakhstan

* Correspondence: danil@njfu.edu.cn

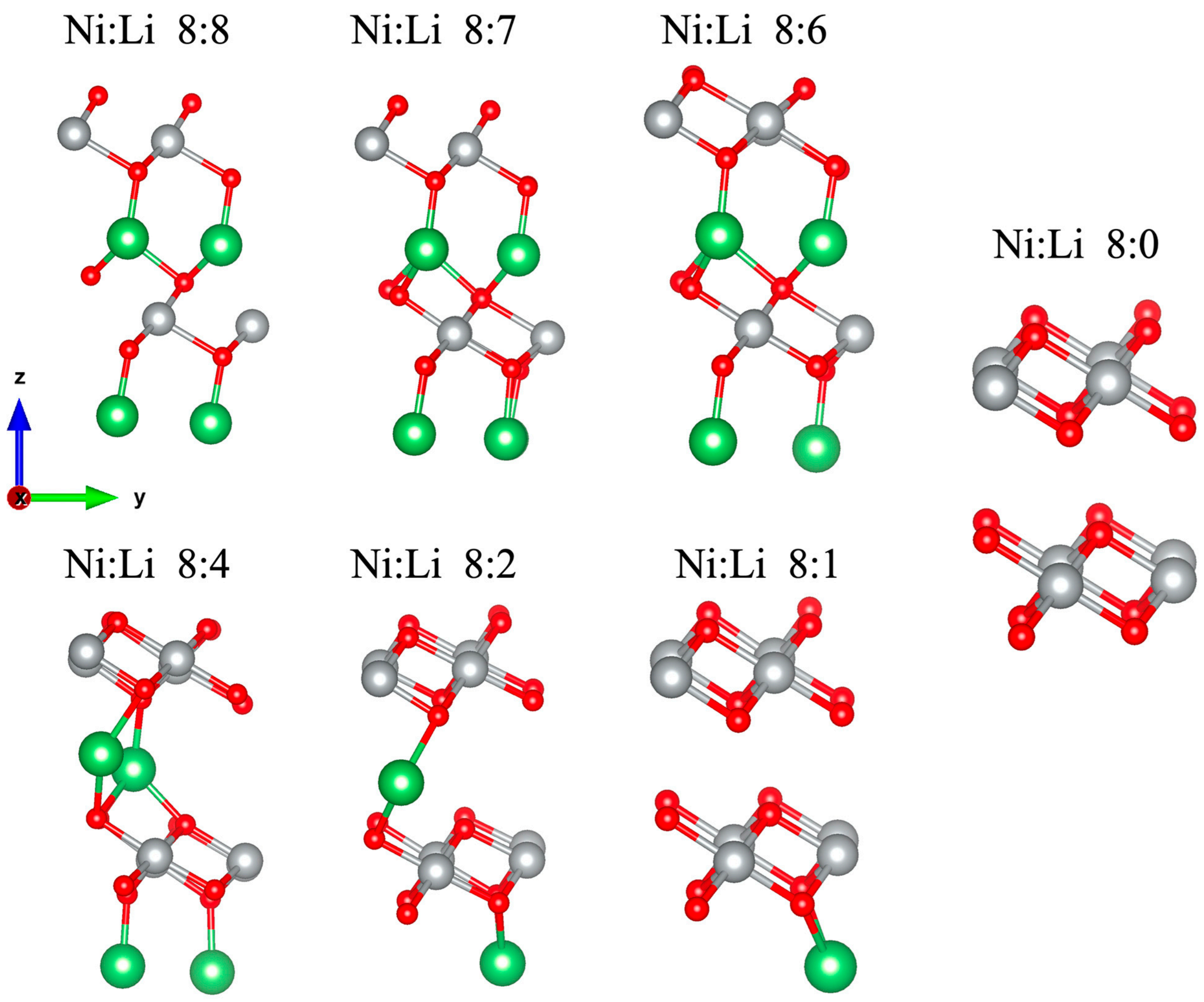

Figure S1. Optimized atomic structure of the NiO_2 supercell at different Li loadings. Nickel atoms are shown in grey, oxygen in red, and lithium in light green.

Table S1. Dataset for Figure 3a.

Impurities concentration	0	12.5	25	37.5	50
Black solid	0.850905	0.959195	1.02533	1.14241	1.57073
Black dotted	0.833176	0.937621	1.14814	1.58545	2.09928
Red Solid	0.850905	0.866544	0.860966	0.852994	0.84337
Red dotted	0.833176	0.843177	0.842986	0.833319	0.835905
Blue solid	0.850905	-	0.923971	-	0.991968
Blue dotted	0.833176	-	0.930495	-	1.00271

Table S2. Dataset for Figure 3b.

Impurities concentration	0	12.5	25	37.5	50
Black solid	28.2796122	20.3216	18.2901028	0.0889000000	0.002
Black dotted	28.2796122	20.7246252	22.6179764	16.241	6.246
Red Solid	28.2796122	58.3808594	133.0023934	201.5225497	340.187
Red dotted	28.2796122	19.8918632	103.31208	164.83496494	144.3556319
Blue solid	28.2796122	-	112.8739	106.08108508	197.47882679
Blue dotted	28.2796122	-	115.7529558	120.558	231.85259246

Table S3. Dataset for Figure 3c.

Impurities concentration	0	12.5	25	37.5	50
Black solid	1.68461581	1.70814381	1.80595039	1.543498	1.38479674
Red Solid	1.68461581	1.52763482	1.45533641	1.20067	-2.17567279
Blue solid	1.68461581	-0.01592397	-	-0.122	-0.30392818

Table S4. Dataset for Figure 4c.

Li removed	Black Solid	Black Dotted	Green Solid	Green Dotted	Blue Solid	Blue Dotted	Red Solid	Red Dotted	Cyan Solid	Cyan Dotted
0	1.0788259496	1.0787286752	1.0847371184	1.0948716355	1.1005706578	1.1059259528	1.1099048877	1.0958070564	1.088605849	1.0909792886
12.5	1.0788259496	1.0849743571	1.0992415862	1.1053424323	1.1000820753	1.1084002839	1.099851854	1.1246551916	1.104134503	1.1032561488
25	1.0818236437	1.0790937861	1.0899920395	1.0812117995	1.0945597955	1.096921527	1.1072089185	1.1700209721	1.1176399225	1.1166496368
37.5	-	-	1.1031279694	1.0766077269	1.0890575856	1.1137988135	1.104105503	1.0936651146	1.1174067133	1.1371143462
50	1.100934285	1.1114091718	1.1192189568	1.1499768094	1.0913901217	1.0998780976	1.1081731217	1.0771048185	1.1268	1.1382733396
75	1.0997235041	1.0646724691	1.0290596932	1.2056054585	1.0331523399	1.0561914235	1.1813722697	1.21389	1.183476408	1.2007323578
87.5	0.9910402193	0.988958664	0.992659161	0.9721456550000000	0.9649053167	0.947059467	1.0113365352	0.9623	1.0325102691	1.12252
100	0.8787565617	0.8595022599	0.9317260938	0.8471623218	0.8442303551	0.8298260201	0.9120511686	0.897343	0.9001800558	1.058

Table S5. Dataset for Figure 4d.

Li removed	Black line	Green line	Blue line	Red line	Cyan line
0	1.62826272	1.55466401	1.82433106	1.18575081	2.43848911
12.5	0.34564617	2.12429525	-0.10907595	1.57477201	1.89878263
25	1.6512	-0.40677423	0.98871456	2.09389612	1.22592957
37.5	-	-0.55484831	-0.21070725	1.59262732	1.07399132
50	0.85801611	0.35602488	3.62118877	2.74773815	0.81231848
75	1.0936935	-0.21376888	1.62655565	-0.06403628	1.35168469
87.5	1.33733818	1.08392149	-0.38207595	1.80536192	1.83288084
100	1.33733818	1.43401528	1.895976	1.49470501	1.68771022
